# Diphtheria and Sewer-Gas

**Published:** 1896-01

**Authors:** W. M. Yandell

**Affiliations:** State Quarantine Officer, El Paso, Texas


For the Texas Medical Journal.

### DIPHTHERIA AND SEWER-GAS.

BY DR. W. M. YANDELL, STATE QUARANTINE OFFICER, EL PASO, TEXAS.

FROM April 14th, 1892, to September 15th, of the same year, diphtheria prevailed in El Paso to an extent never before or since known in the city, and being then the city health officer, to whom all cases of contagious diseases are reported, to be at once isolated and quarantined, I was more than ever impressed with the universal popular dread of sewer-gas, and with the commonly received opinion that it was the most frequent cause of diphtheria. That diphtheria is often contracted from sewer-gas I do not believe; that it is ever so contracted I most seriously doubt; the virulence of its contagion, like that of small-pox, is sufficient to account for its spread, without attributing any share of its ravages to sewer-gas. This proposition I can demonstrate, as to this section of the country, to any unprejudiced person. The following tables, covering the period above referred to, are made up from my notes taken at the time: Number of cases: April, 15; May, 27; June, 15; July, 27; August, 6; September, 3; total, 93.

Nationality: Mexicans, 53; Americans, 9; negroes, 2; total, 25. Class of dwellings: Adobe, 26; frame, 20; brick, 17; total, 63.

| Class of Privies. | Mexican. | American. | Negroes. | Total. |
|---|---|---|---|---|
| Sewered.............. | 5 | 8 | 0 | 13 |
| Vaults................ | 5 | 13 | 1 | 19 |
| Boxes................ | 1 | 1 | 2 | 4 |
| Nothing ............ | 26 | 0 | 1 | 27 |
| Grand total.................................63 | | | | |

To sum up (1). Of the 63 places but 13, or about one-fifth, had sewer connection. The sewer system was planned and put in operation by the distinguished sanitary engineer and author, Col. Geo. E. Waring, Jr., famous for having devised the Memphis system and others, and now Street Cleaning Commissioner of New York City, and is of vitrified earthenware pipe, the greater part of which is only six inches in diameter, the main sewer being only fourteen inches, and with every house connection ventilated by a four inch cast iron pipe. The flow of sewage is rapid and uniform and it never stagnates in the pipes.

(2) The Mexican inhabitants are nearly all poor, and the greater part of them live in two settlements almost exclusively their own, and outside of the sewer system; of the thirty-seven houses twenty-six had no privies, and the inmates of those houses, as a rule, defecate on the surface of the ground and cover their excrement with dry earth. While not a particularly cleanly people as to their persons, no very poor people are, as a rule, their houses are generally well ventilated and quite neat internally, and their refuse is exceedingly small. In this land of sunshine and dry air, the small amount of waste matter thrown out by them is rapidly dried and rendered innocuous.

(3) In the most complete sewered part of the city, almost entirely occupied by American residences, only 5 of the 93 cases occurred.

Again: Socorro, Texas, sixteen miles below El Paso, with a population of eight hundred, all Mexicans, is a typical Mexican village. It covers at least four square miles of territory; in fact is practically a country settlement, the families widely scattered, yet, in the spring of 1891, there were 23 deaths there from diphtheria in one month.

Fort Hancock Station, fifty miles below El Paso, had a population, in 1891, of something over two hundred, nine-tenths Mexicans. Nine children died there of diphtheria in two weeks in that year. The sanitary surroundings of the place are remarkable. It is situated in what might be called a desert of sand, so that in November, 1892, just after the cholera scare, when Dr. Jenkins, then Health Officer of the Port of New York, Dr. Chas. S. Roberts, Chief Inspector of Contagious Diseases, Dr. Nagle, of the Health Department, both of New York, and Dr. A. N. Bell, the distinguished editor of the *Sanitarian*, stopped there for a few minutes, Dr. Jenkins observing the entire absence of any apparent cause for the spread of disease, remarked:

"Cholera couldn't exist here," and the others agreed with him. I could multiply instances, every Mexican town on this border furnishing them, even the scattered population of the mountains of West Texas and New Mexico not being excepted.

The poorer Mexicans take no precautions to prevent the spread of the disease, visiting with their children the families in which it prevails, and the contagiousness explains its ravages. Their scattered villages and small amount of waste, purified by sunshine and the dry aseptic atmosphere of this section, absolutely preclude the filth theory.

Moral: Cleanliness is next to godliness, and as hard to attain; the only effect of teaching that diphtheria comes from filth, is to cause people to disregard the danger of contagion, and to keep an eagle eye, not on their own, but to their neighbors' backyards.

